# Supplementary material for: Effect of mitoTEMPO on Redox Reactions in Different Body Compartments upon Endotoxemia in Rats
Source: Biomolecules. 2023 May 5;13(5):794. doi: 10.3390/biom13050794 (PMC10216200; doi:10.3390/biom13050794)
Supplement: Supplementary file 1 [file biomolecules-13-00794-s001.zip › biomolecules-2269490-supplementary.pdf]

Supplementary Figure S1

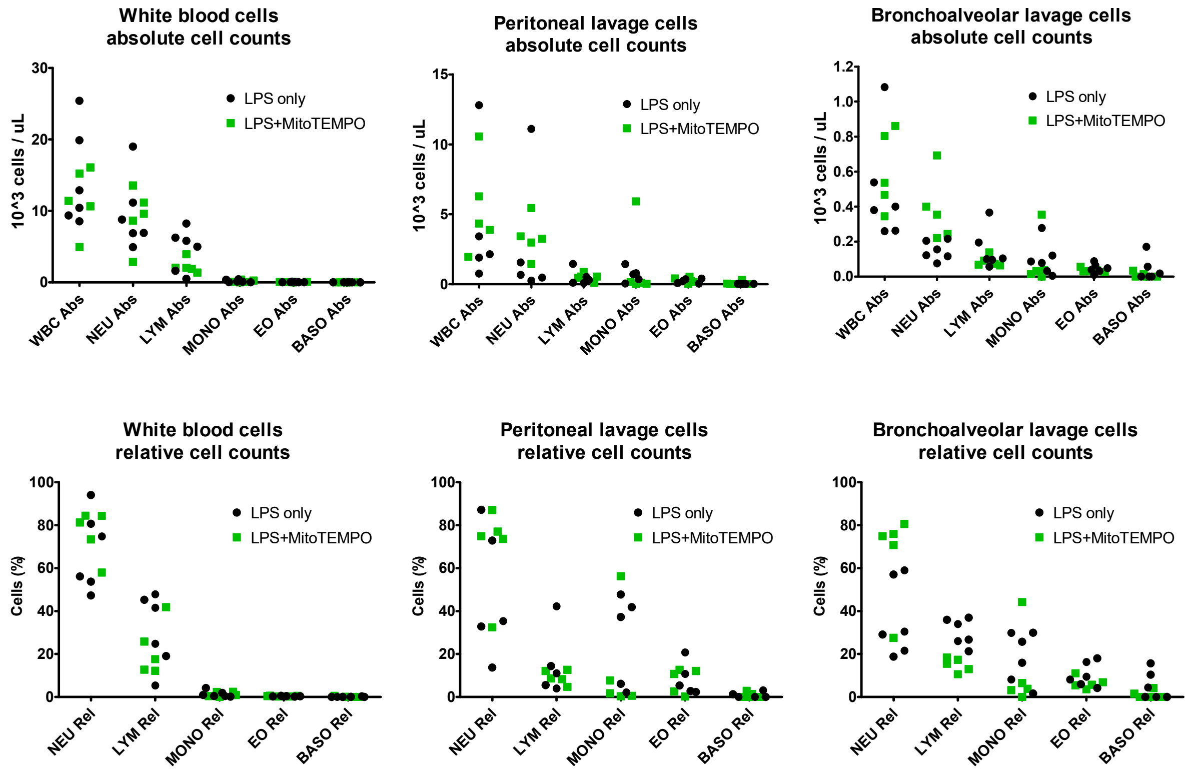

Supplementary Figure S1. Effect of mitoTEMPO on cell counts in blood, and peritoneal and bronchoalveolar fluids.
